# The structure and reactivity of the HoxEFU complex from the cyanobacterium *Synechocystis* sp. PCC 6803

**DOI:** 10.1074/jbc.RA120.013136

**Published:** 2020-05-14

**Authors:** Jacob H. Artz, Monika Tokmina-Lukaszewska, David W. Mulder, Carolyn E. Lubner, Kirstin Gutekunst, Jens Appel, Brian Bothner, Marko Boehm, Paul W. King

**Affiliations:** 1 Biosciences Center, National Renewable Energy Laboratory, Golden, Colorado, USA; 2 Department of Chemistry and Biochemistry, Montana State University, Bozeman, Montana, USA; 3 Botanical Institute, Christian-Albrechts-University, Kiel, Germany

**Keywords:** *Synechocystis*, HoxEFU, diaphorase, photosynthesis, kinetics, protein cross-linking, electron paramagnetic resonance (EPR), bidirectional hydrogenase, protein-protein interaction, nickel-iron enzyme, cooperativity, hydrogenase, nickel

## Abstract

Cyanobacterial Hox is a [NiFe] hydrogenase that consists of the hydrogen (H_2_)-activating subunits HoxYH, which form a complex with the HoxEFU assembly to mediate reactions with soluble electron carriers like NAD(P)H and ferredoxin (Fdx), thereby coupling photosynthetic electron transfer to energy-transforming catalytic reactions. Researchers studying the HoxEFUYH complex have observed that HoxEFU can be isolated independently of HoxYH, leading to the hypothesis that HoxEFU is a distinct functional subcomplex rather than an artifact of Hox complex isolation. Moreover, outstanding questions about the reactivity of Hox with natural substrates and the site(s) of substrate interactions and coupling of H_2_, NAD(P)H, and Fdx remain to be resolved. To address these questions, here we analyzed recombinantly produced HoxEFU by electron paramagnetic resonance spectroscopy and kinetic assays with natural substrates. The purified HoxEFU subcomplex catalyzed electron transfer reactions among NAD(P)H, flavodoxin, and several ferredoxins, thus functioning *in vitro* as a shuttle among different cyanobacterial pools of reducing equivalents. Both Fdx1-dependent reductions of NAD^+^ and NADP^+^ were cooperative. HoxEFU also catalyzed the flavodoxin-dependent reduction of NAD(P)^+^, Fdx2-dependent oxidation of NADH and Fdx4- and Fdx11-dependent reduction of NAD^+^. MS-based mapping identified an Fdx1-binding site at the junction of HoxE and HoxF, adjacent to iron-sulfur (FeS) clusters in both subunits. Overall, the reactivity of HoxEFU observed here suggests that it functions in managing peripheral electron flow from photosynthetic electron transfer, findings that reveal detailed insights into how ubiquitous cellular components may be used to allocate energy flow into specific bioenergetic products.

*Synechocystis* sp. PCC 6803 has long served as a model photosynthetic organism to address questions on the biochemistry and mechanisms of photosynthetic electron transfer, including the function of peripheral redox enzymes that maintain redox homeostasis (1, 2). In *Synechocystis* 6803, one example is the bidirectional hydrogenase HoxEFUYH, which, depending on the metabolic context, either functions to dispense with excess reducing power through the generation of hydrogen or couples the oxidation of hydrogen to the reduction of electron carrier pools (3, 4). HoxEFUYH has long been of interest due to the possibility of engineering the enzyme and metabolic pathways to enable photobiological hydrogen production routes (5). Despite numerous investigations, the underlying features of the biochemistry, structure, and protein-protein interactions of HoxEFU have yet to be fully detailed. A more complete understanding of these properties could be used to improve approaches for engineering photobiohydrogen production, artificial photosynthesis, or catalyst design.

HoxEFUYH consists of the small and large [NiFe] hydrogenase subunits, HoxY and HoxH, respectively, and an additional set of subunits that compose the heterotrimeric diaphorase HoxEFU (6) (Fig. 1). Biochemical reactions with ferredoxins 1 and 4 (Fdx1 and Fdx4; *ssl0020* and *slr0150*, respectively), flavodoxin (Fld), and NAD(P)H demonstrate that each of these was capable of supporting catalytic H_2_ production by Hox in cell extracts (4, 7, 8) and purified enzyme (9) (Table S1). Studies with the intact HoxEFUYH heteropentamer have further suggested there is a preference for NADH over NADPH (Table S2) (4, 7, 9–12). The ability of HoxEFUYH to couple H_2_ oxidation to NAD(P)^+^ reduction is less clear, however. In cell extracts, this activity was either undetectable (7) or barely detectable (8), and in whole cells where H_2_ oxidation was observed, the electron acceptor was not definitively identified (13). The reactivity of purified HoxEFU for NAD(P)^+^ reduction has not yet been clearly shown and may differentiate *Synechocystis* 6803 HoxEFUYH from other related enzymes that have more readily reversible diaphorase activity (10, 11, 14), and in particular Hox proteins (HoxYH with or without the diaphorase subunit) from nonphotosynthetic bacteria, which can have different reactivities and subunit compositions.

**Figure 1.**
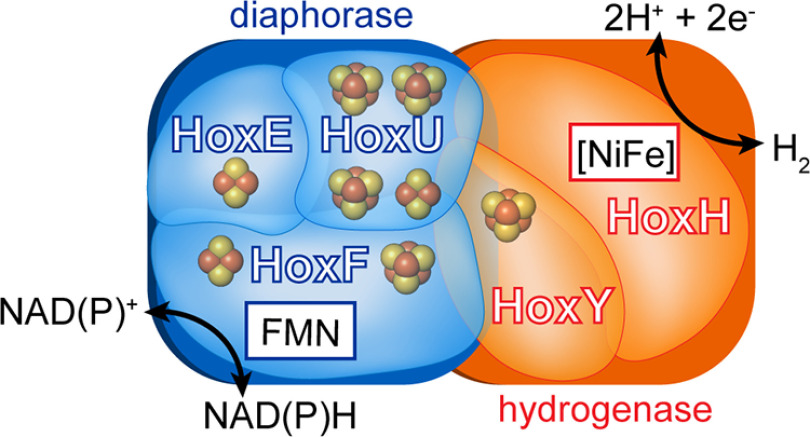
**Scheme of the multimeric HoxEFUYH bidirectional hydrogenase from** *Synechocystis* 6803. The complex is composed of HoxEFU diaphorase (*blue*) and HoxYH [NiFe] hydrogenase (*orange*) subunits containing a flavin (FMN) cofactor and [NiFe] active site, respectively, along with multiple iron-sulfur clusters (sphere representation).

**Table 1:** **Reaction kinetics of *Synechocystis* 6803 HoxEFU**

Reaction	*K_m_* and *K*′ (µM)	*k*_cat_ (s^−1^)	*k*_cat_/*K_m_* (M^−1^·s^−1^)	*V*_max_ (µmol min^−1^ mg^−1^)
NADH oxidation to MB reduction	39 (*K_m_* for NADH)	4.25	1.1 × 10^5^	2.5
NADPH oxidation to MB reduction	1003 (*K_m_* for NADPH)	9.69	9.6 × 10^3^	5.7
NADH oxidation to Fld reduction	ND*^a^*	0.005	ND	0.003
NAD^+^ + Fld_red_	ND	0.003	ND	0.002
NAD^+^ + Fdx1_red_	15.4 (*K*′ for Fdx1_red_)	0.63	4.1 × 10^4^	0.37
NADP^+^ + Fdx1_red_	59 (*K*′ for Fdx1_red_)	3.8	6.4 × 10^4^	2.2

*^a^* ND, not determined.

**Table 2:** **Reactivity of *Synechocystis* 6803 HoxEFU with Fdx proteins**

Fdx	NAD^+^ reduction (*k*_obs_, s^−1^)	NADH oxidation (*k*_obs_, s^−1^)	*E_m_* (mV *vs.* NHE)
Fdx1 (*ssl0020*)	0.63	Not observed	−412 (25)
Fdx2 (*sll1382*) (28)	Not observed	0.02	−246 (28)
Fdx4 (*slr0150*) (27)*^a^*	0.81	Not observed	−440 (30)*^b^*
Fdx5 (*slr0148*) (27)	Not observed	Not observed	Not determined
Fdx11 (*ssl3044*)*^c^*	0.10	Not determined	Not determined

*^a^* Previously assigned as Fdx3 (4).

*^b^* *E*_m_ value of the Fdx1 homologue from *T. elongatus* (30).

*^c^* Fdx11 was identified in this work via CyanoBase (72).

The HoxEFU subcomplex contains a complex array of redox cofactors. Whereas HoxYH harbors the [NiFe] catalytic site with one additional [4Fe-4S] cluster, HoxEFU contains an FMN cofactor in HoxF, as well as seven FeS clusters (three [2Fe-2S] clusters and four [4Fe-4S] clusters) (8). EPR and FTIR spectroscopies have been employed for biophysical characterization of *Synechocystis* 6803 HoxEFUYH and have revealed some unusual characteristics compared with standard [NiFe] hydrogenases (15).

During purification HoxEFUYH is known to readily dissociate to form HoxEFU subcomplexes separate from the HoxYH hydrogenase subcomplex (16–19). This has raised the question of whether the HoxEFU subcomplex has a functionally distinct role in the cell (16, 20). To date, HoxEFU has been observed *in vivo* (16) as well as *in vitro* following isolation of the pentameric complex, exemplified by the observation that in thylakoid membrane imaging, ∼23% of HoxF is found incorporated into the HoxEFU subcomplex rather than the complete HoxEFUYH complex (20).

The details of the fundamental biochemistry of *Synechocystis* 6803 HoxEFU, its biophysical properties, substrate preferences, reaction kinetics, and structural features, specifically the role of HoxE in mediating reactions with soluble electron carriers, are unresolved. HoxE in *Synechocystis* 6803 is known to be required for H_2_ production by Hox coupled to both NADH and NADPH (7) and has been implicated in binding and electron transfer with Fdx, and it is also known to facilitate association of Hox with the thylakoid membrane (20). Investigations with *Thiocapsa roseopersicina* have further supported the role of HoxE in electron transfer (21). Thus, the HoxE subunit is required for several important functions of photosynthetic Hox complexes, though specific features that contribute to diaphorase activity are not completely known. The Hox from *Cupriavidus necator*, in contrast, reversibly catalyzes the oxidation of NADH in the absence of HoxE, although it does include the nonhomologous HoxI. Unlike HoxE, HoxI does not bind an FeS cluster (12, 17) and is therefore thought not to play a role in electron transfer. The Hox complex from *Hydrogenophilus thermoluteolus* catalyzes H_2_ oxidation coupled to NAD^+^ reduction and has neither a HoxE nor a HoxI subunit (22). Thus, how subunit compositions are linked to biochemical functions across Hox diversity and the role of individual subunits remain to be resolved.

To address questions about the relationship of structural and compositional properties of *Synechocystis* 6803 Hox to its biochemical function, the *Synechocystis* 6803 HoxEFU was isolated as an intact subcomplex, and the biophysical properties, reactivity, and structural composition were determined. The results demonstrate that HoxEFU cooperatively couples NAD(P)H oxidation and reduction (diaphorase activity) to the exchange of electrons with other redox carriers, including Fdx proteins and Fld, all in the absence of the HoxYH hydrogenase subcomplex. The biochemical and structural features identified here demonstrate that HoxEFU has the essential biochemical properties to catalyze redox reactions with photosynthetic electron transfer components in a more nuanced and dynamic manner than previously established.

## Results and discussion

### Initial expression and characterization of HoxEFU

Heterologous expression of HoxEFU in *Escherichia coli* was carried out based on the procedure previously described for HoxEFUYH in *Synechocystis* 6803 (15). The expression construct included a Strep-II tag on the C terminus of HoxF and conserved ribosomal binding sites from the *hox* operon in *Synechocystis* 6803. Affinity purification under anaerobic conditions yielded ∼1.5 mg of HoxEFU per liter of growth medium, and MS was used to verify subunit composition (Fig. S1) and flavin incorporation. FMN incorporation was verified through UV-visible spectroscopy, with a ratio of 0.7 FMN per HoxEFU.

### EPR spectroscopy of HoxEFU with natural electron donor-acceptors

**Figure 2.**
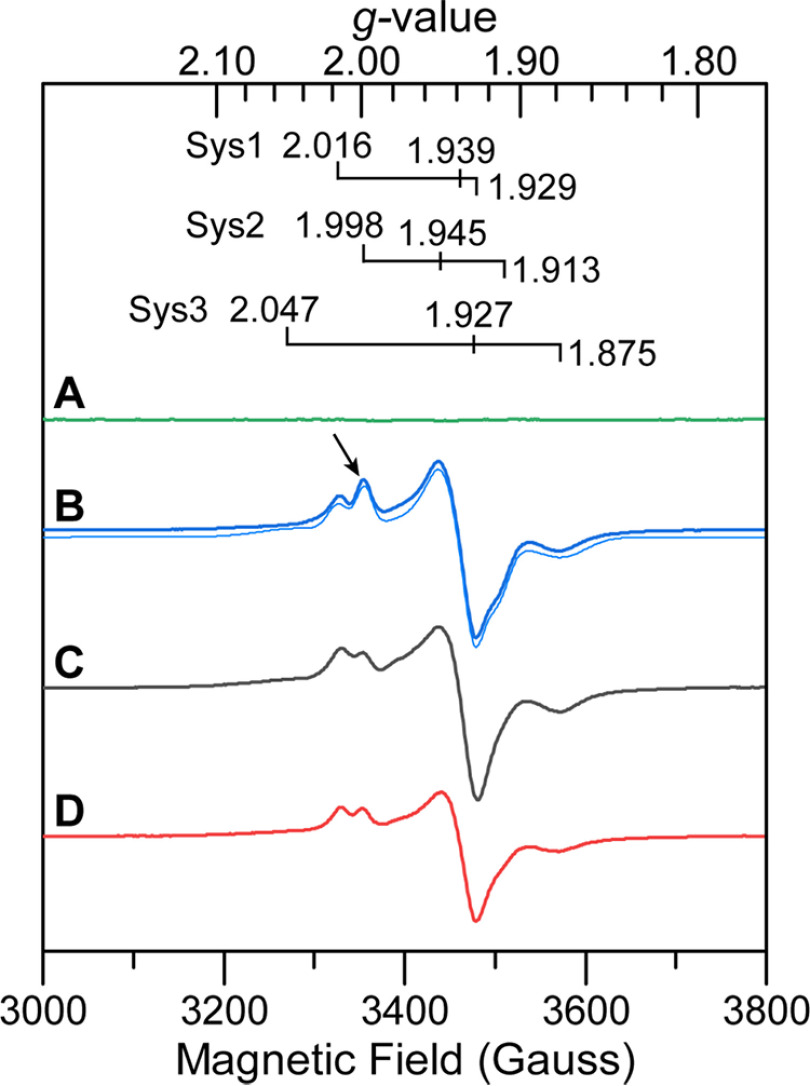
**Continuous-wave X-band EPR of HoxEFU prepared under different reduction-oxidation conditions.** *A* as purified. *B*, *dark blue line*, reduced with 20 mm DT; *light blue line*, simulated spectra with the individual spin systems (Sys) specified above. *C,* reduced with 10 mm NADH. *D,* reduced with 10 mm NADPH. For all samples, HoxEFU (50 μm) was prepared in Tris buffer at pH 8.3. The EPR spectra were collected at 15K and 1 mW microwave power.

To determine whether HoxEFU can react with NAD(P)H and mediate electron transfer reactions via the FeS clusters, the effect of NAD(P)H on the reduction-oxidation state of the HoxEFU cofactors was determined by EPR spectroscopy. The anaerobically as-purified HoxEFU was EPR silent (Fig. 2, *green spectrum*) due to the lack of added reductant (such as NAD(P)H or sodium dithionite (DT)) during the purification process. To determine the spectrum of the reduced HoxEFU complex, the as-purified sample was reduced with DT (midpoint redox potential (*E_m_*) = −660 mV *versus* normal hydrogen electrode (NHE), pH 7 (23)). This resulted in a spectrum with an overall rhombic line shape that accounted for 6.5 ± 1.5 spins mol^−1^ (Fig. 2, *blue spectrum*), compared with the expected count of 7 spins mol^−1^ for full incorporation of FeS clusters.

Reduction of HoxEFU by the physiological donors NAD(P)H yielded spectra similar to that of the DT-treated sample, with 5.3 ± 1.5 spins mol^−1^, indicating nearly complete reduction (Fig. 2, *black and red spectra*) and confirming the ability of the pyridine nucleotides to react with FMN. The slightly lower spin concentration compared with the DT-treated sample likely results from one of the FeS cofactors not being fully reduced by NAD(P)H, evidenced by a less intense spectral feature at a g value of 2.0 (see feature highlighted with an arrow in Fig. 2*B* compared with Fig. 2, *C* and *D*) in NAD(P)H- *versus* DT-reduced HoxEFU. Interestingly, reduction of HoxEFU by NAD(P)H was more effective in reducing the accessory clusters (∼5 spins mol^−1^) than HoxEFUYH at 1.9 spins mol^−1^ (15). This most likely is due to HoxEFUYH being under turnover conditions for H_2_ production (unlike HoxEFU, which cannot catalyze reduction of protons), which could prevent the observation of fully reduced FeS clusters under steady-state conditions.

All reduced samples showed strong intensity near the middle of the spectrum (g = 1.93) and broad features at the wings (most prevalent at low temperature). The broadening likely reflects magnetic coupling between the multiple reduced FeS clusters, consistent with their function in electron transfer for HoxEFU (15, 24). Several features at the low- and high-field regions of the spectrum showed similarity to the FeS cluster signals reported for the HoxEFUYH pentameric complex (15), and the signals could be further resolved based on temperature and microwave power dependence (Fig. S3). This includes slower relaxing signals (optimal temperature, 25 K) at g values of 2.00, 1.94, and 1.90 and faster relaxing signals (optimal temperature, 4 K) at g values of 2.05, 1.98, and 1.88, which are consistent with [2Fe-2S] and [4Fe-4S] clusters, respectively. To further corroborate these assignments, EPR simulations were carried out on the DT-reduced HoxEFU sample (Fig. S3). Unlike the prior report on HoxEFUYH (15), the simulations did not readily converge when only two spin systems were included; however, the addition of a third system was able to account for the measured EPR signal. The simulations include two systems (g = 2.16, 1.939, and 1.929, and g = 1.998, 1.945, and 1.913) attributable to [2Fe-2S] clusters based on the temperature relaxation properties, which were in close agreement with the g values in the prior report, and one broad system (g = 2.047, 1.927, and 1.875) that accounts for nearly half the spin density in the overall signal (Fig. S3). The latter can be assigned to a [4Fe-4S] cluster type signal based on the temperature relaxation properties, though the greater weight of this signal in the simulation indicates that it may have overlapping contributions from additional clusters; alternatively, the greater weight may be a result of a different effect, such as power saturation at low temperature. The simulations begin to distinguish the various FeS cluster components of the overall signal, although full deconvolution is difficult due to the rich FeS cluster content of the protein. It should also be noted that the FMN semiquinone is not directly observed under NAD(P)H or DT reduction; rather, it may be in the fully reduced hydroquinone and EPR-silent form or obscured by overlapping signals in the g = 2 region.

**Figure 3.**
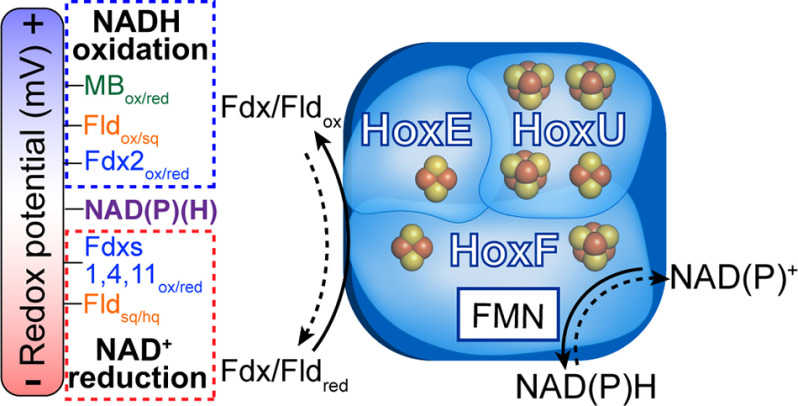
**Model of *Synechocystis* 6803 HoxEFU reactivity.** HoxEFU catalyzes diaphorase reactions either accepting electrons from lower-potential donors, such as Fdx_red_ or Fld_red_ (*dotted red box*), or donating electrons to higher-potential acceptors, such as Fdx2_ox_ or Fld_ox_ (*dotted blue box*).

### Reaction kinetics of HoxEFU with NAD(P)H, ferredoxin, and flavodoxin

The HoxEFUYH complex couples the oxidation of reduced electron carriers to the production of H_2_. HoxEFU is required for the reactivity with electron carriers; however, the binding site interactions and kinetics are not well known. To determine the reaction kinetics and preferences of HoxEFU for different electron carriers, we measured the reactivity in coupled reactions with redox dyes. The oxidation of NAD(P)H by HoxEFU (Table 1) is a half-reaction of the NAD(P)H-to-H_2_ reaction catalyzed by HoxEFUYH, with *k*_cat_/*K_m_* values of 1.1 × 10^5^ and 9.6 × 10^3^ for NADH and NADPH, respectively. The *K_m_* value of HoxEFU for NADH of 39 μm is within the previously reported values of 12–83 μm for the intact HoxEFUYH complex (8, 10), whereas the measured HoxEFU *K_m_* value for NADPH of 1 mm is ∼10-fold greater than was previously reported for H_2_ production by *Synechocystis* 6803 cell extracts (8). This may reflect differences in assay conditions, or differences in the binding and reactivity of HoxEFU for NADPH in the absence of HoxYH. Previous reports on Hox activity measured H_2_ evolution or uptake on cell extracts (4, 7, 10, 11) or used electrochemical methods with purified enzymes where the quantity of electroactive enzyme was not defined (6, 14). As such, it is not possible to directly compare specific rates obtained here on purified HoxEFU with those previously reported results.

The NAD(P)H measurements employed coupled reactions using the redox dye methylene blue (MB; *E_m_* = +11 mV *versus* NHE), rather than the natural substrates (*i.e.* Fdx). To assess kinetics of a physiologically relevant reaction, HoxEFU activity was measured for NAD(P)^+^ reduction coupled to Fdx1_red_ (*E_m_* = −412 mV *versus* NHE (25)) oxidation. Unlike the dye-based assays, a plot of the reaction velocity indicated cooperative kinetics, with kinetic fits for NAD^+^ and Fdx_red_ having Hill coefficients of 2.8 (Fig. S5; Table S3). The observation of cooperativity suggests that HoxEFU might assemble into oligomers, where binding of Fdx1 or NAD^+^ to one HoxEFU could induce long-range effects on binding of Fdx1 or NAD^+^ at additional HoxEFU binding sites (26). In contrast, NADP^+^ did not exhibit cooperativity with Fdx1_red_, and a higher *K_m_* of 59 μm for Fdx1_red_ was observed. The *k*_cat_/*K_m_* values for both NAD^+^ and NADP^+^ reduction were lower than those for NAD(P)H oxidation (Table 1). The kinetic assays demonstrate a systematic preference for NAD(H) over NADP(H), a trend consistent with prior measurements of NAD(P)H-dependent H_2_ production by HoxEFUYH. (Table S1) (8, 10, 11).

Fld serves as an electron carrier in peripheral photosynthesis pathways and was previously shown to stimulate H_2_ production by HoxEFUYH in whole-cell extracts (4). To assess whether HoxEFU is able to react with Fld as a substrate, the reduction of NAD(P)^+^ with reduced Fld was tested. The redox couples of Fld are approximately −433 and −240 mV *versus* NHE, for the fully reduced hydroquinone and semiquinone, respectively (25). Based on the relative thermodynamic favorability of the NADP^+^/NAD(P)H redox couples (*E_m_* = −320 mV), it seemed reasonable that HoxEFU could mediate Fld-based reduction of NAD^+^, as well as the reverse reaction. Indeed, HoxEFU coupled oxidation of Fld_red_ to the reduction of NAD^+^, albeit at a lower *k*_cat_ than the Fdx1_red_ reaction (0.003 s^−1^ *versus* 0.63 s^−1^) (25). The formation of the Fld semiquinone species was observed concurrently with the formation of NADH. In the reverse direction, HoxEFU oxidized NADH and reduced Fld to the semiquinone state, with a *k*_cat_ of 0.005 s^−1^, after an initial lag period. In contrast to Fdx1, the one-electron reduction of Fld (*E_m_* = −240 mV *versus* NHE) from NAD(P)H oxidation (*E_m_* = −320 mV *versus* NHE) is thermodynamically more favorable. The *k*_cat_ of HoxEFU-mediated reduction of NAD^+^ by oxidation of Fld_red_ was 10^−3^ s^−1^, ∼100-fold lower than the reaction using Fdx1_red_. In cell extracts, reduced Fld stimulated higher H_2_ production rates than Fdx1 (with HoxEFUYH), with a lower apparent *K_m_* than Fdx1 (4). Based on our kinetic results, it is suggested that there might be conformational and/or reactivity differences between HoxEFU and HoxEFUYH that may regulate the substrate preferences and/or reaction kinetics differently in HoxEFU to favor Fdx over Fld.

### Reactivity with additional ferredoxins

The ability of HoxEFU to reduce Fld suggests that other soluble redox carriers with similar midpoint potentials may also serve as redox partners. To test this, diaphorase activity was measured under standardized conditions for four additional Fdx proteins from *Synechocystis* 6803 (Table 2; sequence alignment in Fig. S2), using previously described gene numbering (27). These assays were performed using estimated *V*_max_ conditions of 50 μm Fdx and 2 mm NAD(H), though the *k*_obs_ values may not be equivalent to *k*_cat_.

Fdx2 (*sll138*) has an *E_m_* of −243 mV (28), similar to the *E_m_* of the Fld semiquinone. Notably, Fdx2 has a unique intracellular role compared with Fdx1 in mediating iron homeostasis and chlorophyll accumulation, and it is conserved in photosynthetic microbes (27, 29). While the rate of NADH oxidation to reduction of Fdx2_ox_ was low (*k*_obs_ = 0.02 s^−1^), it was 10-fold higher than that of Fld_ox_. The reverse reaction of NAD^+^ reduction by Fdx2_red_ was not detected.

Fdx4 (*slr0150*) is closely related to Fdx1, although they have different expression and interaction profiles (27). Analysis of the homologous Fdx from *Tricondyloides elongatus* revealed a midpoint potential of −440 mV *versus* NHE (30), and it has been shown to stimulate H_2_ production in *Synechocystis* 6803 HoxEFUYH (4). Thus, it was hypothesized that it would support NAD^+^ reduction by HoxEFU to a similar degree as Fdx1, and indeed this was found to be the case, with a *k*_obs_ of 0.81 s^−1^, slightly higher than that of Fdx1.

Fdx5 (*slr0148*) is found on the same operon as Fdx4 and thus is similarly regulated. However, Fdx5 harbors a [2Fe-2S] cluster, is more closely related to bacterial than plant type Fdx proteins, and is more distantly related to Fdx1 (27). Under the conditions we tested, HoxEFU showed no activity with Fdx5 for either NAD^+^ reduction or NADH oxidation. The lack of activity may be due to some factor present in the cell that was not captured in the *in vitro* assay, such as phosphorylation (31), or it may be a result of inefficient binding and electron transfer between Fdx5 and HoxEFU.

An additional cyanobacterial Fdx, which we named Fdx11 (*ssl3044*), was discovered via CyanoBase and putatively assigned as a [2Fe-2S] cluster Fdx of 10.8 kDa. Potential Fdx11 interaction partners predicted by the STRING database (32) include the photosynthetic components PsbO and PsaF. Fdx11 was found to be able to support the reduction of NAD^+^ to NADH by HoxEFU with a *k*_obs_ of 0.10 s^−1^, consistent with a midpoint potential below that of the NAD^+^/NADH couple. This activity establishes a new reactivity pathway for HoxEFU which may involve photosynthetic electron transfer.

Collectively, the results with the different Fdx proteins demonstrate that HoxEFU has the capacity to react with a range of physiological electron carriers and that the direction of the reaction is highly potential dependent. Size, surface charge, and midpoint potential are all factors that may influence binding and reactivity. The relative contributions of these factors are difficult to determine given that to date, the only Fdx from *Synechocystis* 6803 that has a solved crystal structure is Fdx1 (33). The number of Fdx proteins that can react with HoxEFU implies that Hox functions within a complex reactivity network and suggests the possibility that HoxEFU may react with other electron carriers, such as other ferredoxins, cytochromes, or quinones. The diaphorase activity of HoxEFU, therefore, might function independently of HoxYH and hydrogenase activity to exchange reducing equivalents among the carrier pools in cells. However, this requires further *in vivo* investigation.

The activities measured here demonstrate that the HoxEFU subcomplex has substrate reactivities both complementary to and distinct from the intact HoxEFUYH complex for coupling redox reactions between NAD(P)H, and Fdx or Fld. The fact that HoxEFUYH is further able to couple Fdx, Fld, or NAD(P)H to H_2_ activation implies that it may manage a single redox reaction cycle utilizing 3 redox substrates (4). This reactivity would be similar to the electron bifurcation reaction that has been observed for [FeFe] hydrogenases from anerobic microbes (34, 35). On the other hand, the NAD(P)H-Fdx/Fld linked reactivity of HoxEFU may be specific to the subcomplex to afford flexible channeling of electron flow among substrate pools. It has also been hypothesized that HoxEFU functions to reduce, and thereby reactivate, the oxygen-inactivated [NiFe] site in HoxYH, a hypothesis that is not excluded by our results (6). The ability of HoxEFU to mediate an exchange of electrons between the NAD(P)H, Fdx, and Fld pools are especially interesting in light of the recent discovery that cyanobacterial photosynthetic complex I (NDH-1) exclusively accepts electrons from reduced ferredoxin (36).

### Equilibrium binding isotherms for NAD(P)H

In order to further evaluate the cooperativity observed by the Fdx1/NAD^+^ kinetic assays, we performed FRET studies on HoxEFU, in which the binding interactions of NADH with HoxEFU are monitored by the fluorescence emission. The equilibrium binding isotherm demonstrates that NADH binds cooperatively to HoxEFU, with a Hill coefficient of 1.9 and a *K_d_* of 32 μm, close to the *K_m_* of NADH in the MB reduction reaction (Fig. S4*A*).

**Figure 4.**
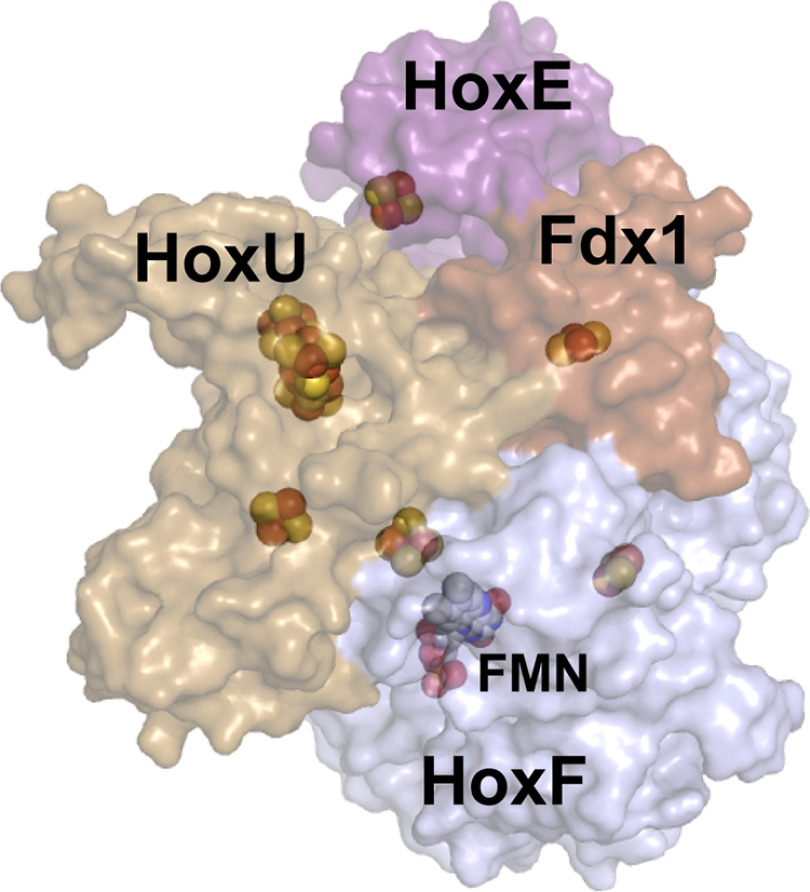
**HoxEFU-Fdx1 binding model based on cross-linking MS and homology modeling of HoxE, HoxF, and HoxU subunits.** Fdx1 (*brown structure*) is modeled to bind in a cleft between the HoxE (*purple structure*) and HoxU (*gold structure*) subunits. The iron-sulfur and flavin (FMN) contents of the individual subunits are depicted as spheres.

We also examined the equilibrium binding isotherms of NAD^+^ and NADP^+^ to HoxEFU. Both pyridine nucleotides showed strong binding cooperativity, with Hill coefficients of 1.77 ± 0.07 and 1.70 ± 0.15 for NAD^+^ and NADP^+^, respectively, and *K_d_* values of 1350 ± 70 and 1950 ± 270 μm (Fig. S3, *B* and *C*; Table S3). Thus, NADP^+^ dissociates from HoxEFU more readily than NAD^+^, consistent with activity measurements that show a preference for NAD^+^ over NADP^+^. The *K_d_* values also reflect pyridine nucleotide concentrations that are likely well above the concentration of ∼80 nmol per g of fresh cell weight previously reported for *Synechocystis* 6803 (37). Based on the cooperativity that was observed, only a fraction of HoxEFU binds with NAD(P)^+^ at concentrations below ∼500 μm, with a steep increase in bound pyridine nucleotide to ∼2 mm. The binding kinetics agree well with the reaction kinetics results and support a function of the HoxEFU subcomplex in catalytic oxidation of NAD(P)H coupled to reduction of soluble carriers, rather than the Fdx-dependent reduction of NAD(P)^+^. Although oxidation of NAD(P)H was observed using MB as an artificial electron acceptor, no oxidation was observed when MB was replaced with Fdx_ox_. This is likely a result of unfavorable thermodynamics of reducing a more negative potential Fdx (Fdx1, *E_m_* = −412 mV *versus* NHE) by the more positive potential of NAD(P)H (*E_m_* = −320 mV) (Fig. 3). Under different intracellular conditions when there is a high concentration of NAD(P)^+^ relative to NADPH, such as in cases of dark-to-light transition (38, 39), or under low light and nitrogen deprivation (40), HoxEFU may catalyze Fdx_red_-dependent reduction of pyridine nucleotides in a reaction similar to that catalyzed by Fdx-NADP^+^ reductase. The *K_d_* results suggest that this is not a primary function of HoxEFU, though it remains a possibility that the addition of the HoxYH subcomplex could modify reactivity.

The demonstration that Fdx1 can donate electrons to HoxEFU supports the capacity of HoxEFUYH to mediate Fdx1-dependent evolution of H_2_ (4), perhaps without a requirement for NAD(P)H oxidation. An additional scenario is that HoxEFUYH is capable of a coupled reaction such as electron bifurcation, which is suggested by the relative midpoint potentials of HoxEFUYH's redox cofactors. Indeed, the low *k*_cat_/*K_m_* value of 10^4^ identified for HoxEFU in the Fdx1-dependent reduction of NADP^+^ may be an outcome of a short-circuited bifurcation reaction in the absence of HoxYH. To our knowledge there are no reports in the literature of assays that have measured HoxEFUYH bifurcation.

It is an intriguing observation that the NADP^+^-based kinetics demonstrate a standard Michaelis-Menten-type hyperbolic curve, while the equilibrium binding isotherms show sigmoidal Hill-type behavior. This suggests the possibility that binding is indeed cooperative but that the NADP^+^ reduction reaction is observed only once the binding sites are saturated (41, 42). Prior work on glucokinase has shown that addition of a second substrate changes the observed kinetics from sigmoidal to hyperbolic, in a sense masking cooperativity (43, 44). Kinetics are known to differ from equilibrium binding particularly in situations such as when substrate binding is slow or rate-limiting (45). Furthermore, the observation of hyperbolic kinetics does not exclude the reaction from involving a cooperative mechanism (46).

### Identification of the Fdx1 binding site by MS

The binding site of Fdx1 on Hox has been implicated to involve the HoxE subunit, though the biochemical evidence for a specific binding interaction is lacking (4, 20). The binding interaction of Fdx1 with Hox has important contributions to the control of reaction cooperativity and the electron flow within Hox during turnover. To address this, chemical cross-linking experiments between purified Fdx1 and purified Hox, along with LC-MS/MS, were undertaken to identify potential binding sites of Fdx1 to HoxEFU (Table S4). The identified cross-links place Fdx1 in a cleft between HoxE, HoxF, and HoxU (Fig. 4). Additional Fdx1-HoxEFU cross-links that were identified may provide alternative binding configurations or be an outcome of allosteric or oligomeric (monomer or dimer) state changes in HoxEFU.

Our cross-linking MS results support the role of Fdx1 in binding simultaneously to HoxE, HoxF, and HoxU, allowing electron transfer from Fdx1 to the [2Fe-2S] cluster in either HoxE or HoxF, with the branched arrangement of FeS clusters in HoxU allowing for multiple possible electron transfer routes between the active site and other interaction partners. It is important to note that HoxU shows a strong structural similarity to the N-terminal part of [FeFe] hydrogenases, which holds its four accessory FeS clusters. Since [FeFe] hydrogenases are well-known Fdx-oxidizing enzymes, one of the FeS clusters of HoxU might be an electron recipient in the HoxEFU module as well as in HoxEFUYH. The structural model most closely related to *Synechocystis* 6803 Hox is from *Hydrogenophilus thermoluteolus*; it lacks HoxE and has a HoxF with significant sequence differences from *Synechocystis* 6803 HoxF. Thus, the *Synechocystis* 6803 HoxEFU-Fdx1 model obtained here represents a significant advance in understanding the site of binding of Fdx1 to *Synechocystis* 6803 HoxEFU. Prior biochemical results on HoxEFUYH from *Synechocystis* 6803 have shown that Fdx1 is a competent electron donor for catalyzing H_2_ production, which may involve Fdx1 binding to the site identified here for HoxEFU, and/or additional binding sites (4).

## Conclusions

Collectively, our results show that HoxEFU is capable of functioning *in vitro,* independently of HoxYH, to couple redox reactions between NAD(P)H, Fdx1, Fdx2, Fdx4, Fdx11, and Fld (Fig. 3). The variability in the efficiencies of different ferredoxins to exchange electrons with NAD(P)H via HoxEFU underpins the idea that the high number of ferredoxins in *Synechocystis* supports a robust network of redox regulation. It remains to be shown if the ability of the diaphorase to shuttle electrons between the NAD(P)H, Fdx, and Fld pools is of physiological importance *in vivo*. We have identified cooperative binding kinetics of HoxEFU, which are important for reactivity with physiological electron donors. Cross-linking MS provides a structural model for how Fdx1 interacts with HoxEFU. The addition of HoxYH may further tune HoxEFU activity by adding electron transfer pathways and possibly modifying the oligomeric state of the Hox complex. Catalytic rates of pyridine nucleotide reduction by HoxEFU *versus* HoxEFUYH suggest that substrate reactivity changes in the presence of HoxYH or other interaction partners. While many of the functions of HoxEFU identified here may be conserved among other Hox proteins, changes to subunit composition such as in *H. thermoluteolus,* which lacks HoxE (22), and *C. necator,* which replaces HoxE with the unrelated HoxI (12), could alter both reactivity and higher-order structure. Our results will enable further efforts to understand the structural and biophysical mechanisms related to biological electron transfer and the role of HoxEFU in photosynthetic processes.

## Experimental procedures

### Protein expression and purification

HoxEFU expression was similar to that previously reported for *Synechocystis* 6803 HoxEFUYH (15). The *Hox* operon was modified to add a Strep-II tag with a serine-alanine linker on the C-terminal end of HoxF. The protein-encoding genes of unknown function were removed from the gene construct, but the intergenic regions encoding for ribosomal binding sites were conserved. The *hoxE*, *hoxF*, and *hoxU* gene sequences were optimized for expression in *Escherichia coli* using GenScript's proprietary software. GenScript synthesized the gene and cloned it into the pET21 vector.

The pET21HoxEF*U vector was transformed into Δ*iscR* Kan^r^ BL21 competent cells. Five fresh colonies were used to inoculate a 150-ml overnight culture in Terrific broth medium, which was grown at 37°C and 225 rpm. After 16 h, 3 ml of the overnight was inoculated into 1 liter of prewarmed Terrific broth medium and grown to an OD_600_ of 0.4. To induce protein expression, the cultures were treated with 1 mm IPTG, supplemented with ferric ammonium citrate (4 mm final), cysteine (2 mm final), sodium fumarate (25 mm final), and FMN (10 μm final), and sparged with argon overnight at room temperature. All subsequent treatments were strictly anaerobic. Cells were harvested by centrifugation at a relative centrifugal force (RCF) of 6037 for 5 min, resuspended in buffer, and frozen at −80°C.

For purification, EDTA-free protease tablets, lysozyme, and DNase were added to thawed cell pellets, which were then lysed by passage through a microfluidizer 10–12 times. The lysate was then centrifuged at an RFC of 149,000 for 1 h, and the clarified lysate was applied directly to a Strep-XTHC column. Elution was performed with 10 mm biotin, with a typical yield of purified protein at 1.5 mg liter of culture^−1^.

HoxEFU protein identification from both in-gel and in-solution digestion was performed according to standard protocols recommended by the manufacturer using a trypsin (Promega) protease:complex ratio of 1:50–1:100 overnight and for 3 h, respectively. Proteins were identified as described elsewhere (47) using a maXis Impact UHR-QTOF instrument (Bruker Daltonics) interfaced with a Dionex 3000 nano-uHPLC (Thermo Fisher) followed by data analysis in Peptide Shaker v.1.13.6 (48). Intact protein analysis was performed as described previously using a Bruker micrOTOF mass spectrometer (Bruker Daltonics) coupled to a 1290 ultrahigh pressure series chromatography stack (Agilent Technologies) (49, 50).

Fdx1 expression was performed by transformation into BL21 chemically competent cells. Five to ten colonies were used to inoculate a 150-ml LB overnight culture, which was shaken at 225 rpm at 37°C. This culture was then used to inoculate 1 liter of LB medium, the cells were grown to an OD_600_ of 0.6, and expression was induced with 1 mm IPTG and supplemented with ferric ammonium citrate (4 mm final) and cysteine (2 mm final). After continued growth for 4 h, cells were harvested by centrifugation at an RCF of 6037 for 5 min, resuspended in buffer, and frozen at −80°C. The thawed cell pellet was sparged with argon for 10 min, and all subsequent steps were handled anaerobically. Lysis and purification were carried out as for HoxEFU, yielding ∼4 mg liter of culture^−1^.

### Fdx2, Fdx4, Fdx5 and Fdx11 expression and purification

The genes encoding Fdx2, Fdx4, Fdx5, and Fdx11 from *Synechocystis* sp. PCC 6803 (*sll1382*, *slr0150*, *slr0148*, and *ssl3044*, respectively) were cloned into a modified version of the pRSETA vector (Life Technologies) (51, 52), which led to the expression of an Fdx-TEVcs-GST-His fusion protein with a linker sequence (53) between the Fdx and the GST-His tandem affinity tag. *E. coli* KRX cells (Promega, Germany) were used to overexpress the protein overnight in LB medium at 10°C after induction at an OD_600_ of 0.6 with IPTG and ferric ammonium citrate at final concentrations of 1 mm and 0.05% (w/v), respectively. Cells were harvested, resuspended in lysis buffer (50 mm NaPO_4_ (pH 7.0), 250 mm NaCl) and broken by sonication (Sontrode MS73 (Sonopuls, Bandelin, Germany); 8 repeats of 20 s on (70% cycle, 70% power) and 20 s off). The supernatant obtained after ultracentrifugation was incubated for 1 h at 4°C with Talon cobalt affinity chromatography resin (Takara, Germany). Following the incubation period, the resin was washed with 20 column volumes of lysis buffer. Protein elution was performed with 2 column volumes of elution buffer (50 mm NaPO_4_ (pH 7.0), 250 mm NaCl, 500 mm imidazole), and the eluted proteins were dialyzed overnight in 25 mm Tris (pH 7.0)–50 mm NaCl in the presence of 20 mg TEV-His (His tag purified from pRK193 (Addgene, USA)) (54). The following day, the protein was incubated with Talon cobalt affinity chromatography resin. The flow-through was concentrated and loaded onto a HiLoad^TM^ 26/60 Superdex^TM^ 75 prep grade (GE Healthcare, Germany) following the purification method developed by Peden *et al*. (55).

### Flavodoxin expression, purification, and biochemical assays

Flavodoxin (*isiB*) from *Synechococcus* sp. PCC 7002 was recombinantly expressed in *E. coli* BL21(DE3) as described previously (56). Cells were broken with a French press and spun at 35,000 rpm for 1 h at 4°C. The resulting supernatant was loaded onto a DE-52 anion exchange column equilibrated with 50 mm Tris (pH 8), then washed with a gradient of 50–100 mm NaCl, and finally eluted with 500 mm NaCl. Colored fractions were collected, washed with 50 mm Tris (pH 8)–20 mm NaCl, and concentrated by ultrafiltration. An extinction coefficient of 9500 M^−1^ cm^−1^ at 467 nm was used to determine flavodoxin concentration.

Biochemical assays were performed using flavodoxin in a 115-fold excess to HoxEFU, in the presence of 2 mm NADP(H). Reduction of flavodoxin by HoxEFU was monitored by following the decrease of absorbance at 467 nm and concurrent increase at 580 nm. To assay activity in the reverse direction, flavodoxin was reduced with a 100-fold molar excess of dithionite and thoroughly buffer exchanged. Oxidation of the fully reduced flavodoxin by HoxEFU was measured by the increase at 580 nm. Assays were initiated by the addition of NAD^+^ or NADH, and the reaction was monitored at 340 nm for the NADH signal and at 467 and 580 nm for the oxidized and semiquinone species of flavodoxin, respectively.

### Biochemical assays

Kinetics were measured using a Cary 4000 UV-visible spectrometer in kinetics mode. For pyridine oxidation the reaction was initiated by the addition of MB and the reaction monitored at 666 nm. For pyridine reduction, reduced ferredoxin was added, and the pyridine signal at 340 nm was monitored. Curves were fit in OriginPro 2019. To prepare the reduced ferredoxin, the ferredoxin was treated with DT in an ∼100-fold molar excess, and the buffer was exchanged repeatedly via centrifugal filters. Removal of dithionite was verified by UV-visible spectroscopy at 316 nm. Activity measurements of Fdx2, Fdx4, Fdx5, and Fdx11 were carried out using 0.3 μm HoxEFU, 50 μm Fdx, and 2 mm NAD(H).

NAD(P)H equilibrium binding kinetics were performed using FRET similarly to previously described assays (57, 58). Fluorescence data were collected at an excitation wavelength of 285 nm and emission from 300 to 500 nm, with slit widths of 8 nm (Fluorolog 3; Horiba). Intrinsic protein fluorescence emission at 348 nm was used to calculate the binding isotherm.

### EPR spectroscopy

Continuous-wave X-band EPR spectroscopy was carried out on a Bruker Elexsys E-500 spectrometer equipped with a helium cryostat and an Super High Q resonator in conjunction with a MercuryITC temperature controller. Spectra were collected at a frequency of 9.38 GHz, power and temperature as noted in the figure legends, modulation frequency of 100 kHz, and modulation amplitude of 10 GHz. Data were baseline corrected as needed with a user-defined function in OriginPro 2019. Simulations were carried out using the “pepper” function in EasySpin 5.2.25 (59). Spin quantitation was performed using the double-integrated EPR spectra and referenced to copper triethylamine samples of known concentration (75 and 100 μm) measured under the same conditions.

### Cross-linking and protein modeling

Protein-protein interactions within the HoxEFU complex and HoxEFU trimer with Fdx1 were examined using chemical cross-linking (60). Briefly, 1.5 μm HoxEFU was chemically cross-linked with 1 mm bis(sulfosuccinimidyl)suberate (BS3) (Thermo Fisher) in 50 mm HEPES/150 mm NaCl buffer (pH 7.2). The HoxEFU-Fdx1 the complex was established by mixing HoxEFU:Fdx1 in a 1:6 ratio and incubated at room temperature for 2 min. Then the complex was exposed to BS3 for 15, 30, or 60 min at room temperature. All cross-linking reactions were quenched by adding Tris HCl (pH 8) to a final concentration of 120 mm. After 15 min of incubation, the resulting mixtures were separated by SDS-PAGE (4–20% linear gradient minigel; Bio-Rad) and stained with Coomassie Brilliant Blue (Thermo Fisher). Next, the entire gel lanes between 15 and 100 kDa (according to the broad-range marker migration profile; Bio-Rad) were digested with trypsin, and the generated peptides were analyzed as described before (47). Cross-linked species were identified using Spectrum Identification Machine (SIM, v.1.2.2.2) (61) and MetaMorpheus (version 0.0.301) as described previously (62).

HoxEFU protein homology models were generated by Phyre2 (63), and energy-minimized models were docked using ClusPro2 with restrictions derived from cross-linking experiments (64–67). Ligand binding site prediction was run in 3DLigandSite (68). The FMN and iron sulfur cluster cofactors were docked using PatchDock (69, 70) for individual subunits, compared with prediction based on 3DLigandSite, and eventually added as rigid bodies to the final HoxEFU-Fdx1 complex model. Molecular graphics were created using PyMOL (71).

## Data availability

All data are contained within the manuscript.

## Supplementary Material

Supporting Information
