# Supplementary material for: The structure and reactivity of the HoxEFU complex from the cyanobacterium Synechocystis sp. PCC 6803
Source: J Biol Chem. 2020 May 14;295(28):9445–54. doi: 10.1074/jbc.RA120.013136 (PMC7363133; doi:10.1074/jbc.RA120.013136)
Supplement: Supporting Information [file supp_295_28_9445__index.html]

The structure and reactivity of the HoxEFU complex from the cyanobacterium Synechocystis sp. PCC 6803 — Diaphorase activity of HoxEFU — The structure and reactivity of the HoxEFU complex from the cyanobacterium Synechocystis sp. PCC 6803 — Diaphorase activity of HoxEFU — Supporting Information 

# The structure and reactivity of the HoxEFU complex from the cyanobacterium *Synechocystis* sp. PCC 6803

## Supporting Information

- Supporting Information (to be published online) - Supporting figures and tables
