# Supplementary material for: The structure and reactivity of the HoxEFU complex from the cyanobacterium Synechocystis sp. PCC 6803
Source: J Biol Chem. 2020 May 14;295(28):9445–54. doi: 10.1074/jbc.RA120.013136 (PMC7363133; doi:10.1074/jbc.RA120.013136)
Supplement: Supporting Information [file supp_RA120.013136_158976_2_supp_528423_qm6rj3.docx]

The structure and reactivity of the HoxEFU complex from the cyanobacterium *Synechocystis* sp. PCC 6803

Jacob H. Artz, Monika Tokmina-Lukaszewska, David W. Mulder, Carolyn E. Lubner, Kirstin Gutekunst, Jens Appel, Brian Bothner, Marko Boehm, Paul W. King

**Supporting Information**

**Contents:**

Figures S1-S4

Supplementary Figure S1. Intact protein mass spectrometry analysis of purified HoxEFU

Supplementary Figure S2. Alignment of *S.* 6803 Fdxs 1, 2, 4, 5, and 11

Supplementary Figure S3. EPR Temperature, Power Series and simulation data

Supplementary Figure S4. Equilibrium binding isotherms

Supplementary Figure S5. Kinetic summary of HoxEFU

Tables S1-S4

Supplementary Table S1. List of reactions observed in *S.* 6803 Hox proteins

Supplementary Table S2. Reported kinetic parameters from various HOXs

Supplementary Table S3. Hill Coefficients for HoxEFU substrates

Supplementary Table S4. Spatial restraints for HoxEFU-Ferredoxin docking


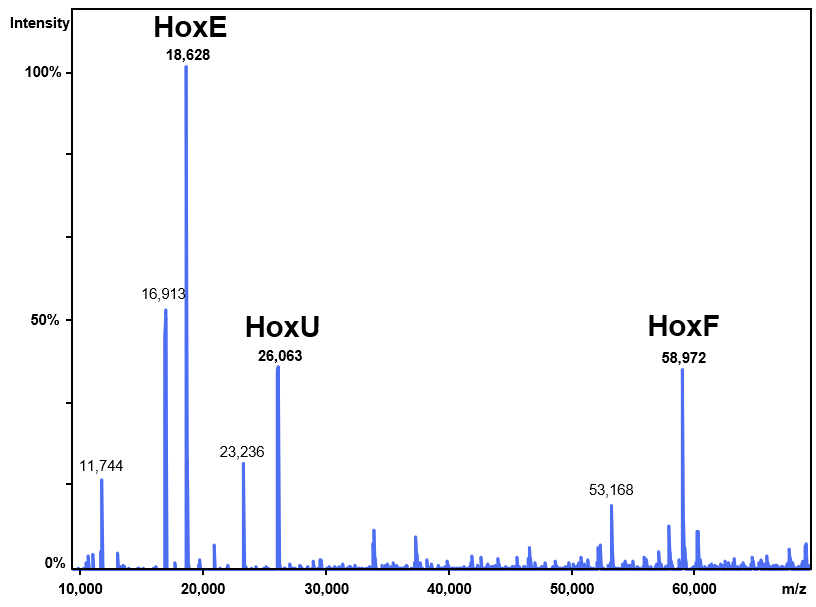


**Figure S1**. Intact protein mass spectrometry analysis of purified HoxEFU. As-purified HoxEFU complex was separated on a reverse phase column in denaturing conditions before infusion to the mass spectrometer. In order to obtain information about exact mass of the intact subunits, the charge state distribution for each unfolded HoxEFU protein was deconvoluted using a maximum entropy algorithm. Based on the mass difference, unlabeled peaks were assigned to be C- or N-terminal truncated versions of HoxEFU subunits.


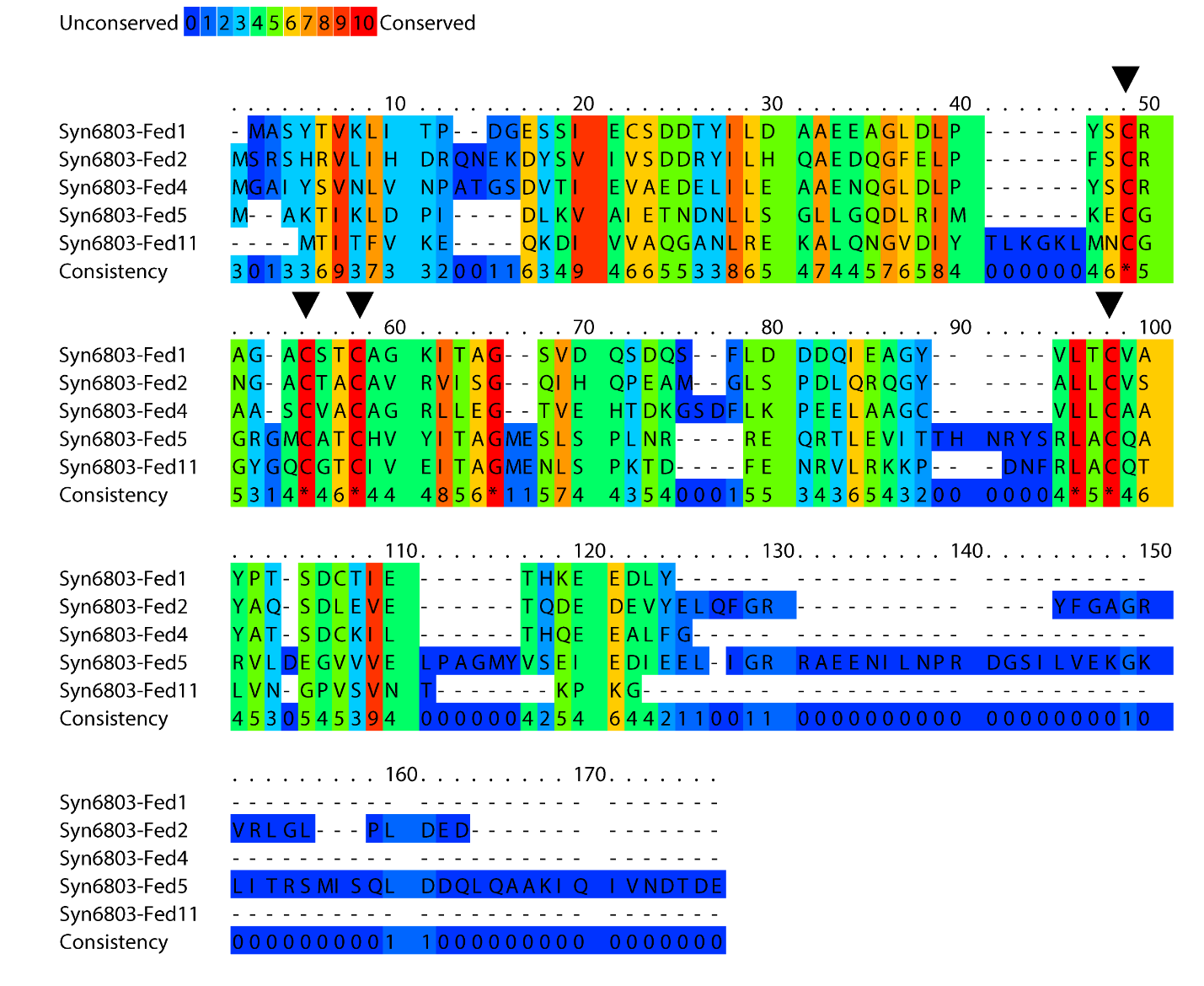


**Figure S2.** Alignment of *S.* 6803 Fdxs 1, 2, 4, 5, and 11. Conserved FeS cluster-coordinating cysteines are indcated by black arrows.


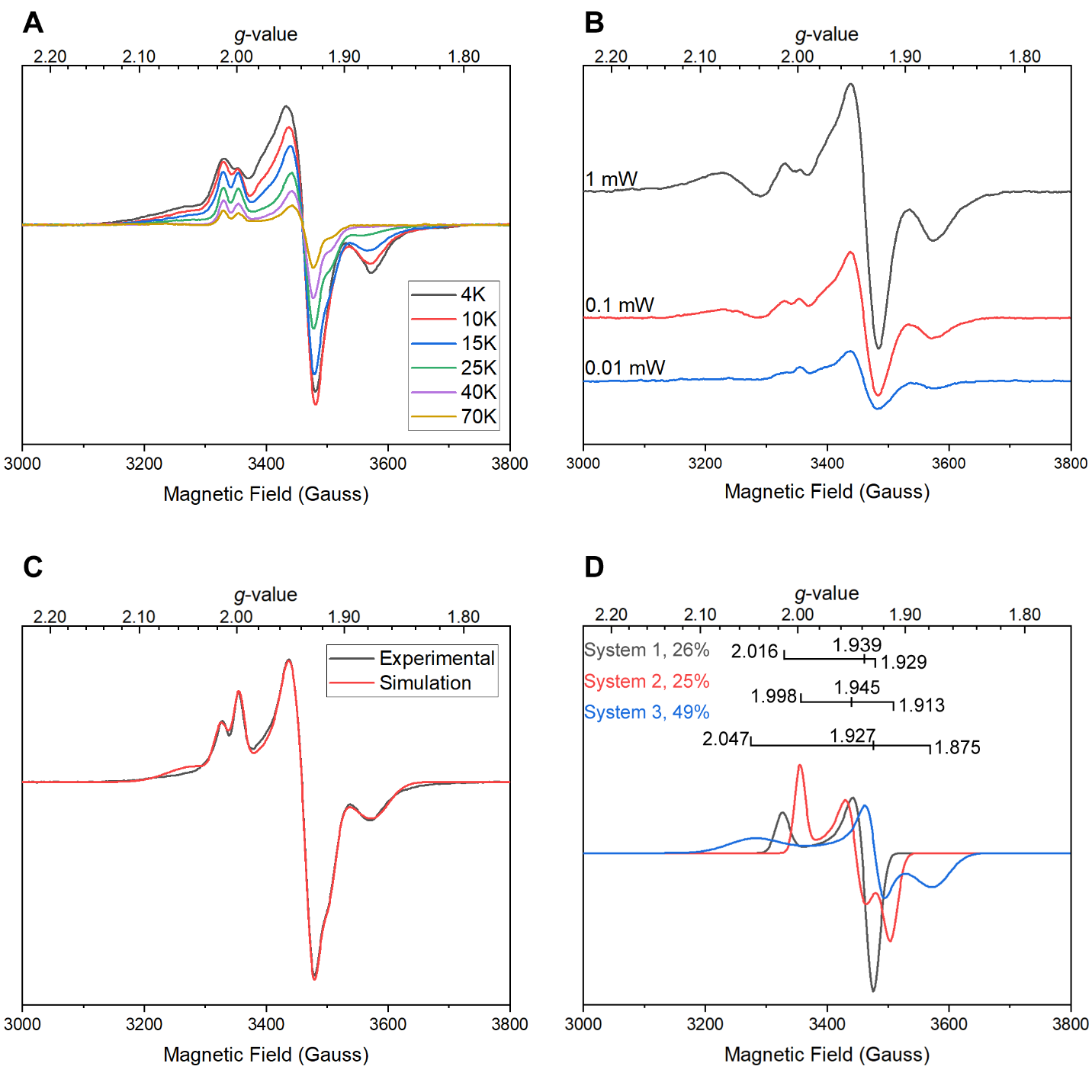


**Figure S3.** EPR temperature and power series of reduced HoxEFU and simulated data. A, temperature series of HoxEFU reduced with 10 mM NADH (measured at 0.1 mW microwave power). B, Microwave power series of DT reduced HoxEFU (measured at 5K). C, Full simulation of DT reduced HoxEFU (black, experimental; red, simulation). D, *g*-value components and relative weights of the simulated signals contributing to the overall EPR simulation in C. The greater weight along with the broader features of system 3 may be reflective of overlapping contributions from additional clusters or spin interactions between FeS clusters. It should be noted that the latter is not specifically accounted for in these simulations and it is possible that other phenomena such as power saturation at low temperature could also contribute to these features.


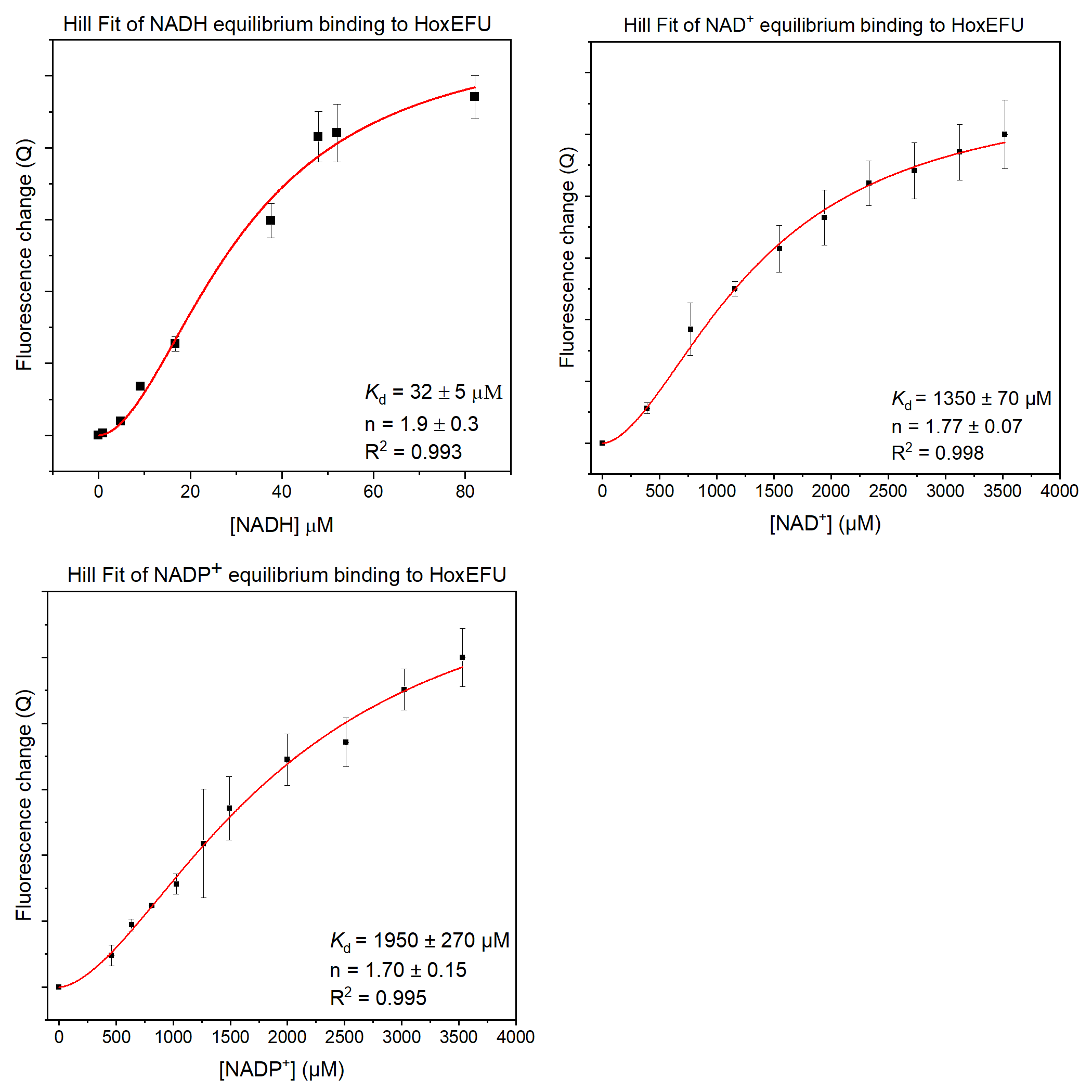


**Figure S4.** Equilibrium binding isotherms of NADH, NAD^+^, and NADP^+^.


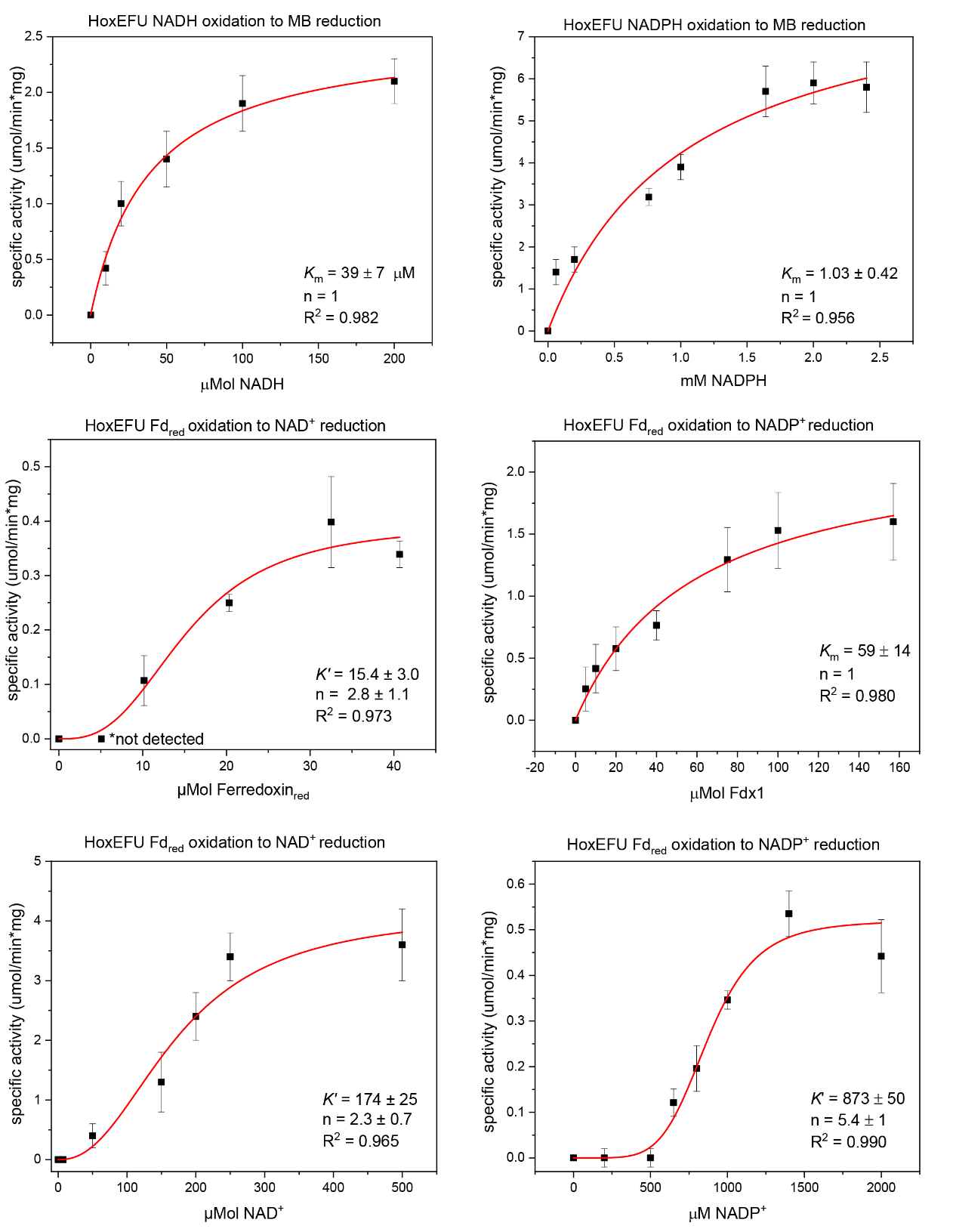


**Figure S5.** Kinetic assays of HoxEFU with NAD(P)(H) and either MB or Fdx1.

**Table S1.** List of reactions observed in *S.* 6803 HOX proteins^†^

| Reaction tested with *S.* 6803 Hox | Subunit composition | Activity observed? | Assay type | Refs |
| --- | --- | --- | --- | --- |
| H_2_ uptake | EFUYH | Yes | Whole cell, acceptor unclear | (1) |
| H_2_+NAD^+^ 🡪 NADH | EFUYH | Yes | Cell extracts | (2) |
| H_2_+NADP^+^ 🡪 NADPH | EFUYH | No | Extract | (2) |
| Fdx1_red_ 🡪 Fdx1_ox_ + H_2_ | EFUYH | No | Purified | (3) |
| Fld_red_🡪 Fld_ox_ + H_2_ | EFUYH | Yes | Cell extracts | (4) |
| Fdx1_red_🡪 Fdx1_ox_ + H_2_ | EFUYH | Yes | Cell extracts | (4) |
| NADH 🡪 NAD^+^ + H_2_ | EFUYH | Yes | Cell extracts  Purified protein | (3,5) |
| NADPH 🡪 NADP^+^ + H_2_ | EFUYH | Yes | Cell extracts  Purified protein | (3,5) |
| H_2_+ NAD^+^ $\rightleftharpoons$NADH | FUYH | No | Cell extracts | (2) |
| H_2_+ NADP^+^ $\rightleftharpoons$NADPH | FUYH | Yes | Cell extracts | (2) |
| Fdx1_red_ + NAD^+^🡪 Fdx1_ox_ + NADH | EFU | Yes | Purified | This work |
| Fdx1_red_ + NADP^+^🡪 Fdx1_ox_ +NADPH | EFU | Yes | Purified | This work |
| NAD(P)H +Fdx1_ox_🡪 NAD(P)^+^ + Fdx1_red_ | EFU | No | Purified | This work |
| NAD(P)H + MB_ox_🡪 NAD(P)^+^ + MB_red_ | EFU | Yes | Purified | This work |
| NADH + Fld_ox_ 🡪 NAD^+^ + Fld_red_ | EFU | Yes | Purified | This work |
| NAD^+^ + Fld_red_ 🡪 NADH + Fld_ox_ | EFU | Yes | Purified | This work |
| NADH + Fdx2_ox_ 🡪 NAD^+^ + Fdx2_red_ | EFU | Yes | Purified | This work |
| NAD^+^ + Fdx2_red_ 🡪 NADH + Fdx2_ox_ | EFU | No | Purified | This work |

**^†^**Additional studies not specified here have also shown H_2_ evolution via dye-coupled assays.

**Table S2:** Reported kinetic parameters from various HOXs

|  | *Gloeocapsa alpicola* HoxEFUYH, purified protein (6) | | *Anacystis nidulans* HoxEFUYH cell extract, H_2_ evolution/uptake activity (nmol/min*mg) (7) | *S.* 6803 HoxEFUYH | | | | *Ralstonia* HoxFU, purified protein *K*_m_ (µM) (8) | *Ralstonia* HoxFUHYI turnover, purified protein (S^-1)^  (9) | *Hydrogenophilus thermoluteolus* purified protein(10) | |
| --- | --- | --- | --- | --- | --- | --- | --- | --- | --- | --- | --- |
|  | H2 evolution/uptake activity (µmol/min per mg protein) | *K*_m_  (µM) |  | H_2_ evolution, purified protein (μmol H_2_ min^−1^ mg^−1^ ) (3) | cell extract  μmol of H_2_ mg Chl^−1^ h^−1^ (5) | cell extract *K*_m_  (µM) (5) | H_2_ evolution, cell extract nmol/(min*mg) (2) |  |  | Specific activity (µmol/min per mg protein) | *K*_m_  (µM) |
| NADH oxidation | 2.3 | 83.0 | 0.098 | 2.8 | 0.09 | 12 | 0.48 | 56 | 222 | 64.9 (to BV) | 1170 |
| NADPH oxidation | 0.7 | 476.0 | 0.035 | 1.4 | 0.09 | 100 | 0.08 | Not reported | 2 |  |  |
| NAD^+^ reduction (H_2_) | 3.5 | 110.0 | 0.18 |  |  |  |  | 197 | 143 | 52.7 | 469 |
| NADP^+^ reduction (H_2_) | 1.1 | Not determined | 0.075 |  |  |  |  |  | No activity could be determined |  |  |

**Table S3.** Hill Coefficients for HoxEFU kinetics and equilibrium binding assays.

| **Kinetic conditions** | | | | **Equilibrium binding conditions** | | | |
| --- | --- | --- | --- | --- | --- | --- | --- |
|  | Hill coefficient | R^2^ of curve fit | *K_m_* or *K’* (µM) |  | Hill coefficient | R^2^ of curve fit | *K*_d_ (µM) |
| **NADH/MB** | 1 | 0.982 | 39 ± 7 | **NADH** | 1.9 ± 0.3 | 0.992 | 32 ± 5 |
| **NADPH/MB** | 1 | 0.956 | 1030 ± 420 | **NADPH**^a^ | ND | ND | ND |
| **NAD^+^ / Fdx1_red_** | 2.8 ± 1.1 (Fdx1)  2.3 ± 0.7 (NAD^+^) | 0.973  0.965 | 15.4 ± 3.0 (Fdx1)  174 ± 25 (NAD^+^) | **NAD^+^** | 1.77 ± 0.07 | 0.998 | 1350 ± 70 |
| **NADP^+^ / Fdx1_red_** | 1 (Fdx1)  5.4 ± 1  (NADP^+^) | 0.980  0.990 | 59 ± 14 (Fdx1)  873 ± 50 (NADP^+^) | **NADP^+^** | 1.70 ± 0.15 | 0.995 | 1950 ± 270 |

^a^ Elevated concentrations of NADPH required for FRET introduced high background fluorescence.

**Table S4**. Spatial restraints for HoxEFU-Fdx1 complex docking

| **Restraint Set** | **Required percent of groups** | **Residue 1**  **HoxF** | **Residue 2**  **Fdx1** | **Min. distance**  **Å** | **Max. distance**  **Å** |
| --- | --- | --- | --- | --- | --- |
| 66% | 100% | 12 | 39 | 1 | 10 |
|  | 100% | 532 | 3 | 1 | 15 |
|  | 100% | 511 | 82 | 1 | 10 |
|  |  | 462 | 46 | 1 | 10 |
|  |  | 182 | 46 | 1 | 10 |
|  |  | Residue 1  HoxF | Residue 2  HoxU |  |  |
| 80% | 80% | 302 | 194 | 1 | 20 |
|  |  | 12 | 42 | 1 | 20 |
|  |  | 501 | 69 | 1 | 20 |
|  |  | 456 | 131 | 1 | 20 |
|  |  | Residue 1  HoxFU-Fdx | Residue 2  HoxE |  |  |
| 83% | 83% | Fdx92 | 120 | 1 | 20 |
|  |  | HoxF194 | 31 | 1 | 20 |
|  |  | HoxF276 | 67 | 1 | 20 |
|  |  | Fdx46 | 21 | 1 | 20 |
|  |  | HoxF316 | 86 | 1 | 20 |
|  |  | HoxU132 | 39 | 1 | 20 |

To identify the Fdx1 binding site on HoxEFU we undertook cross-linking-LC-MS/MS experiments on mixtures of purified HoxEFU and Fdx1. The results identified multiple cross-linking sites that were distributed over the surface of the HoxEFU complex. Most of the sites were localized on HoxF, with fewer connections on HoxE and HoxU. The restraint-based docking solution with the lowest free-energy score is displayed in Fig. 4.

**References**

1. Appel, J., Phunpruch, S., Steinmüller, K., and Schulz, R. (2000) The bidirectional hydrogenase of *Synechocystis* sp. PCC 6803 works as an electron valve during photosynthesis. *Arch. Microbiol.* **173**, 333-338

2. Aubert-Jousset, E., Cano, M., Guedeney, G., Richaud, P., and Cournac, L. (2011) Role of HoxE subunit in *Synechocystis* PCC6803 hydrogenase. *FEBS J.* **278**, 4035-4043

3. Schmitz, O., Boison, G., Salzmann, H., Bothe, H., Schutz, K., Wang, S. H., and Happe, T. (2002) HoxE--a subunit specific for the pentameric bidirectional hydrogenase complex (HoxEFUYH) of cyanobacteria. *Bioch. Biophys Acta* **1554**, 66-74

4. Gutekunst, K., Chen, X., Schreiber, K., Kaspar, U., Makam, S., and Appel, J. (2013) The bidirectional NiFe-hydrogenase in *Synechocystis* sp. PCC 6803 is reduced by flavodoxin and ferredoxin and is essential under mixotrophic, nitrate-limiting conditions. *J. Biol. Chem.*

5. Cournac, L., Guedeney, G., Peltier, G., and Vignais, P. M. (2004) Sustained Photoevolution of Molecular Hydrogen in a Mutant of *Synechocystis* sp. Strain PCC 6803 Deficient in the Type I NADPH-Dehydrogenase Complex. *J. Bacteriol.* **186**, 1737-1746

6. Serebryakova, L. T., and Sheremetieva, M. E. (2006) Characterization of catalytic properties of hydrogenase isolated from the unicellular cyanobacterium *Gloeocapsa alpicola* CALU 743. *Biochem. (Mosc)* **71**, 1370-1376

7. Schmitz, O., and Bothe, H. (1996) NAD(P)^+^-dependent hydrogenase activity in extracts from the cyanobacterium Anacystis nidulans. *FEMS Microbio. Lett.* **135**, 97-101

8. Lauterbach, L., Idris, Z., Vincent, K. A., and Lenz, O. (2011) Catalytic Properties of the Isolated Diaphorase Fragment of the NAD^+^-Reducing [NiFe]-Hydrogenase from *Ralstonia eutropha*. *PLOS ONE* **6**, e25939

9. Burgdorf, T., van der Linden, E., Bernhard, M., Yin, Q. Y., Back, J. W., Hartog, A. F., Muijsers, A. O., de Koster, C. G., Albracht, S. P., and Friedrich, B. (2005) The soluble NAD^+^-Reducing [NiFe]-hydrogenase from *Ralstonia eutropha* H16 consists of six subunits and can be specifically activated by NADPH. *J. Bac.* **187**, 3122-3132

10. Wahlefeld, S. M. (2018) *Activation of H2 and CO2.* Doctoral Thesis, Technische Universität Berlin
